# A 13-Month-Old With Xanthogranulomatous Pyelonephritis With Features of Renal Malakoplakia

**DOI:** 10.1177/2324709616630573

**Published:** 2016-02-05

**Authors:** Tova Appleson, Asma Sharif, Suman Setty, Dennis Liu, Shihtien Wang, Robert Kanard, Kimberly Czech

**Affiliations:** 1 University of Illinois at Chicago, IL, USA

**Keywords:** malakoplakia, xanthogranulomatous pyelonephritis, hydronephrosis, renal mass, anemia, Michaelis-Gutmann

## Abstract

Xanthogranulomatous pyelonephritis is an uncommon chronic inflammatory renal disorder caused by chronic infection with gram-negative bacteria leading to destruction of the renal parenchyma and replacement with foamy lipid-laden macrophages. Renal malakoplakia is another rare form of chronic inflammatory granulomatous disease in the kidney associated with infection usually occurring in adults with immunocompromised status or debilitating disease. It is hallmarked by the finding of foamy histiocytes with distinctive basophilic inclusions (Michaelis-Gutmann bodies). We present a case of a 13-month-old male with history of congenital hydronephrosis who presented with clinical and radiologic findings suggestive of xanthogranulomatous pyelonephritis. However, further pathologic studies revealed the presence of Michaelis-Gutmann bodies, which are pathognomonic for renal malakoplakia. With this case we hope to bring further evidence to support that these two conditions are not mutually exclusive but rather represent two pathologic processes on the same disease spectrum.

A 13-month-old Hispanic male with a past medical history significant for congenital bilateral hydronephrosis with a nonfunctioning right kidney, undescended left testis, and anemia for 2 months presented to the Pediatric Nephrology Clinic for a routine follow-up. History taken that day revealed that the patient had progressive abdominal distention with associated pain and fussiness over the past 2 months. His mother attributed his fussiness to severe constipation, which was made worse by addition of iron supplements he was prescribed 2 months prior for a microcytic anemia with a hemoglobin of 7.9 gm/dL. There were no reported fevers, poor feeding, or concerns about abnormalities in his urine. Medications at time of visit included ferrous sulfate and a multivitamin with iron.

His birth history was significant for prenatally diagnosed mild bilateral hydronephrosis with right-sided hydroureter at 33 weeks gestation. He was born at 36 weeks and required a neonatal intensive care unit stay of 9 days for prematurity and hyperbilirubinemia. There was no family history of renal tumor or renal anomalies. Throughout the first year of life, he was growing and developing normally with no signs or symptoms of UTI (urinary tract infection) and no unexplained fever. He had not been on UTI prophylaxis at any time during the first year of life.

The patient was followed since birth by Pediatric Nephrology for bilateral hydronephrosis with multiple radiologic studies acquired. A summary of the radiologic studies is as follows: the patient had mild bilateral hydronephrosis at birth with a normal voiding cystourethrogram. At 4 months of age, a renal ultrasound showed stable bilateral hydronephrosis with multiple hyperechoic structures that were interpreted by the radiologist as possible renal stones. Hydronephrosis of the right kidney progressed to severe by 7 months of age with suggestion of ureteropelvic junction obstruction and persistence of hyperechoic structures. A renal nuclear scan with lasix (Mag-3 scan) was performed at 9 months of age, which showed a poorly functioning right kidney (3%) with normal function of the left kidney (97%) associated with compensatory hypertrophy by renal ultrasound. The right kidney at that time was assumed to be a dysplastic, nonfunctioning kidney since birth. Serum creatinine was normal.

Physical exam on the day of presentation revealed a fussy infant with vital signs significant for an elevated heart rate of 163, respiratory rate of 32, a blood pressure of 128/86, and temperature of 36.8°C. His abdomen was distended, mildly tender to palpation, firm (but not rigid), and tympanic to percussion without ascites. The abdominal exam was concerning for an abdominal mass. The left testis was palpated in the inguinal canal.

An ultrasound of the abdomen was performed on the day of presentation, which demonstrated an enlarged right kidney at 10.7 cm in length with dysmorphic changes concerning for Wilm’s tumor and a normal left kidney (8.2 cm in length). The patient was admitted for further workup of a renal mass. Laboratory findings on admission showed a normal serum blood urea nitrogen and creatinine, persistence of a microcytic hypochromic anemia (Hgb 7.4g/dL) despite adequate iron supplementation, leukocytosis with a neutrophilic predominance, low iron, and low albumin. Lactate dehydrogenase and ferritin were within normal limits. Urinalysis showed no microscopic blood, moderate leukocyte esterase, negative nitrites, and 18 white blood cells/high-power field. Urine culture and blood cultures were negative on admission. A magnetic resonance imaging (MRI) of the abdomen and pelvis (with and without contrast) showed a severely hydronephrotic right kidney with advanced parenchymal atrophy likely related to ureteropelvic junction obstruction; however, no radiologic structures in the kidney were suspicious for malignancy. There was a complex perinephric fluid collection that extended into the abdominal wall with radiologic findings consistent with abscess formation with significant mass effect on adjacent abdominal structures with extension into the right psoas/iliacus muscles.

This imaging suggested an infectious process, rather than a malignant process, which was surprising in the absence of fever, systemic signs and symptoms of infection, and negative urine and blood cultures. One could only assume that a walled-off abscess occupied the entire right kidney with no communication to the collecting system/bladder. A percutaneous nephrostomy tube was placed that drained purulent, foul-smelling fluid with no urine seen. The fluid grew out 2 species of *Proteus mirabilis.* He continued to remain afebrile and without any clinical signs or symptoms of infection. He was initially treated with continuous nephrostomy tube decompression and oral antibiotics (Augmentin and Cefdinir) for 7 weeks with planned delayed surgical nephrectomy. The decision to delay surgical nephrectomy and continue with a prolonged course of antibiotics was to reduce infectious inflammatory processes that could hinder a stable recovery and reduce the likelihood of wound infection and poor healing process with complications. He was followed weekly in clinic at which time routine cultures (fungal and bacterial) from the nephrostomy tube were sent to follow any species changes and changes in resistance patterns of organisms. However, *Proteus* continued to grow from nephrostomy tube drainage and the resistance pattern remained unchanged throughout the course. At about 4 weeks into outpatient treatment, the collecting system became patent, and he developed positive urine cultures for the first time with *Proteus mirabilis*, which was growing from the nephrostomy tubes as well. He remained afebrile and asymptomatic throughout the 7 weeks of outpatient therapy. Fungal cultures were sent on urine and nephrostomy tube drainage, which were negative.

A right nephrectomy was performed at 7 weeks and he was discharged home after 12 days of hospitalization and was afebrile. In the postoperative period, his renal function remained normal and he had no further infectious complications. The chronic microcytic hypochromic anemia (nadir 7.4 g/dL) that was observed in the 2 months prior to treatment and surgical repair resolved spontaneously after nephrectomy and was thought to be secondary to chronic inflammatory processes in the right kidney.

After nephrectomy the pathologic examination of our patient’s specimen showed a grossly enlarged right kidney (178 g) with a nodular outer surface and attached to a portion of the muscle. The specimen was bivalved to reveal collections of yellow-green purulent material, contained within the renal calyces and scattered focal areas of necrosis shown in Figure 1A. The renal capsule was thickened, fibrotic, and ranging in size from 0.7 to 1.3 cm, and thinning of the renal cortex was grossly noted (Figure 1B). No area of normal or uninvolved renal tissue was apparent and no calculi were grossly identified. As shown in Figure 1B, microscopic examination by hematoxylin and eosin staining revealed significant increase in the size of the renal capsule and a decreased amount of perirenal adipose tissue. As seen in Figure 1C, there is extensive granulomatous inflammation, foamy histiocytes, and marked chronic interstitial inflammation. Detection of granulomas prompted further investigation by Fite and Grocott-Gomori’s methenamine silver special stains, which were negative for acid fast bacilli and fungal elements, respectively. Bacterial colonization, acute abscess, and suppurative inflammation were noted on hematoxylin and eosin sections, as consistent with gross findings. Special stain, calcium von Kossa, was performed which revealed multiple Michaelis-Gutmann bodies–targetoid structures (Figure 1D) and concentric concretions both within the histiocyte cytoplasm as well as in the stroma. The presence of Michaelis-Gutmann bodies, along with granulomatous inflammation, and the presence of foamy histiocytes are characteristic features for the diagnosis of renal malakoplakia.

**Figure 1.**
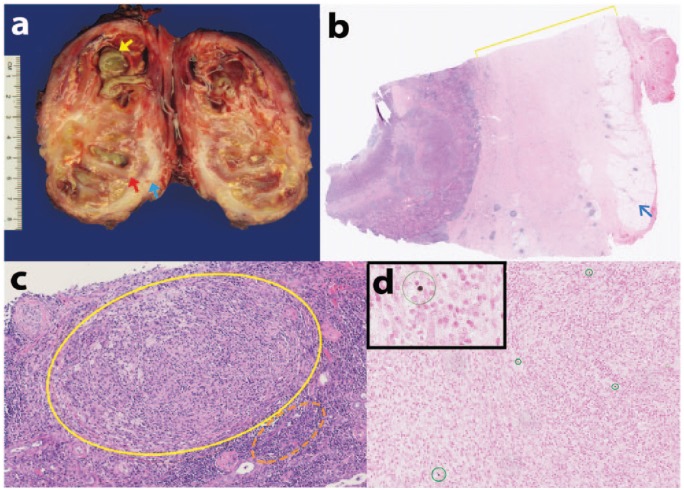
(A) Gross image of the right kidney, bivalved in fresh state: yellow arrow demonstrates collections of yellow-green purulent material in calyces; blue arrow demonstrates thickened white sclerotic renal capsule; red arrow demonstrates thinning of renal cortex. (B) Hematoxylin and eosin stained section of the kidney. Yellow bracket indicates the thickened capsule. Blue arrow indicates decreased perirenal adipose tissue. Original magnification, 25×. (C) Hematoxylin and eosin stained section. Yellow circle indicates granuloma formation with foamy histiocytes; orange dashed-circle indicates interstitial chronic inflammation. Original magnification, 200×. (D) Calcium von Kossa special stain. Insert: Targetoid structure represents a Michaelis-Gutmann body. Multiple concentric concretions in the histiocyte cell cytoplasm and in the extracellular spaces are circled in green. Original magnification, 200×.

## Discussion

In this case, a young child presented with a renal mass that, based on the age, was at high risk for developing a Wilm’s tumor but instead was diagnosed with a chronic granulomatous infection of the kidney due to a congenital renal anomaly. This child lacked all the common symptoms of infection except for an elevated white blood cell count and anemia of chronic disease. It remains speculation whether screening urinalysis or urine culture during routine follow-up in the year prior would have lead to an earlier diagnosis in the absence of overt UTI symptoms. In reviewing the adult literature, UTI was present in about half of the adult cases of xanthogranulomatous pyelonephritis (XGP) at the time of presentation despite a significant renal parenchymal infection that indicates urinalysis is surprisingly not a sensitive screening tool.^1,2^ Of the pediatric case reports described in the literature, abdominal mass (rather than UTI with overt pyelonephritis symptoms) is the presenting sign and would argue against the practice of routine urine screening for these conditions.^3^

Initial imaging was with renal ultrasound followed by MRI to further delineate whether the mass was solid, cystic, or mixed. MRI imaging did not support findings of a solid renal mass such as Wilm’s tumor but instead was consistent with significant urinary tract obstruction, inflammation with parenchymal destruction. Nephrostomy tube placement relieved the obstructive process but based on imaging, renal recovery was not possible due to parenchymal destruction from granulomatous inflammation and ongoing infection, which could not be cleared with medical therapy. Consistent with this idea was compensatory hypertrophy of the remaining, normal left kidney, indicating that the right side was not functional for a long period of time. Therefore, medical treatment versus surgical nephrectomy would have not been beneficial in this case. Indeed, nephrectomy is the procedure of choice for XGP and malakoplakia involving the kidney itself, since the granulomatous inflammation causes irreversible changes to the renal parenchyma as well as the risk of sepsis from a chronic infected tissue mass. There are case reports in pediatric patients utilizing laparoscopic approaches and partial nephrectomies when only a small portion of the renal parenchyma is affected.^3,4^ In addition, there are some reports of malakoplakia treated medically when only the lower urinary tract is involved.^5^

Renal malakoplakia is a form of chronic inflammatory granulomatous disease with the pathologic marker of Michaelis-Gutmann bodies, which most often effects the urinary tract system in patients with underlying systemic diseases or an immunocompromised state. Gram-negative bacteria are most often associated with renal malakoplakia with *Escherichia coli* found in more than two thirds of cases. Renal malakoplakia in children is extremely rare.^6^ Michaelis-Gutmann bodies are described as basophilic, concentric inclusions with amorphous material or foci of calcifications.^7^ These structures are thought to represent accumulations of incompletely digested microbes as a result of defective phagocytosis.^7,8^ Renal malakoplakia is a histological diagnosis and has nonspecific radiologic findings. Most patients demonstrate renal enlargement, disorganization of the renal structures, and poor echogenicity of the kidney on ultrasound examination.^9^

XGP is an uncommon chronic inflammatory renal disorder that is characterized by the destruction of the renal parenchyma and its replacement by solid sheets of foamy lipid-laden macrophages. XGP usually develops during a chronic kidney infection associated with urinary tract obstruction. Gram-negative bacteria are also implicated, most commonly *Proteus mirabilis* or *E coli*. XGP can mimic Wilm’s tumor or other neoplastic disorders.^6^ XGP is further divided into 3 stages based on whether it is confined to the kidneys or extends into the surrounding adjacent structures. Ultrasound imaging will reveal enlargement of the entire kidney, hypoechoic renal areas of calyceal dilatation and parenchymal destruction, and the presence of perinephric fluid collections are often noted. A computed tomography scan may reveal intrarenal lesions such as calculi, areas of renal destruction, and abscesses.^10^ Gross examination reveals replacement of renal parenchyma with fatty tissue. Microscopic examination typically demonstrates granulomatous inflammatory infiltrate with foamy histiocytes, some multinucleated giant cells, lymphocytes, plasma cells, and neutrophils, but no Michaelis-Gutmann bodies are seen.^11^

Cases of renal malakoplakia and XGP resemble each other closely, both grossly and histologically. Both disease processes involve granulomatous inflammation causing destruction of renal parenchyma. Therefore, nephrectomy is the treatment of choice for XGP and most cases of malakoplakia. The distinctive feature of malakoplakia is the presence of Michaelis-Gutmann bodies both within the histiocyte cytoplasm and extracellular spaces as was seen on pathology in our case and reported elsewhere in the literature.^7,8,11^ However, despite the presence of Michaelis-Gutmann bodies, clinically and radiographically our patient’s presentation is more consistent with XGP; the bacterial culture revealed *Proteus mirabilis*, he had ultrasound findings consistent with XGP, and destruction of the renal parenchyma with extension into the perirenal tissues with abscess formation. We present this case as further evidence that malakoplakia and XGP are not exclusive diagnoses but are likely 2 separate entities related to each other on a spectrum.^6^
